# Characterization of complex fluvio–deltaic deposits in Northeast China using multi-modal machine learning fusion

**DOI:** 10.1038/s41598-020-70382-7

**Published:** 2020-08-07

**Authors:** Cyril D. Boateng, Li-Yun Fu, Sylvester K. Danuor

**Affiliations:** 1 School of Geosciences, China University of Petroleum (East China), Qingdao, China; 2 Caburu Sciences, P. O. Box MD 2046, Madina, Accra, Ghana; 3 Department of Physics, Kwame Nkrumah University of Science and Technology, Kumasi, Ghana

**Keywords:** Geophysics, Computer science

## Abstract

Due to the lack of petroleum resources, stratigraphic reservoirs have become an important source of future discoveries. We describe a methodology for predicting reservoir sands from complex reservoir seismic data. Data analysis involves a bio-integrated framework called multi-modal machine learning fusion (MMMLF) based on neural networks. First, acoustic-related seismic attributes from post-stack seismic data were used to characterize the reservoirs. They enhanced the understanding of the structure and spatial distribution of petrophysical properties of lithostratigraphic reservoirs. The attributes were then classified as varied modal inputs into a central fusion engine for prediction. We applied the method to a dataset from Northeast China. Using seismic attributes and rock physics relationships as input data, MMMLF was performed to predict the spatial distribution of lithology in the Upper Guantao substrata. Despite the large scattering in the acoustic-related data properties, the proposed MMMLF methodology predicted the distribution of lithological properties through the gamma ray logs. Moreover, complex stratigraphic traps such as braided fluvial sandstones in the fluvio–deltaic deposits were delineated. These findings can have significant implications for future exploration and production in Northeast China and similar petroleum provinces around the world.

## Introduction

The era of easily discoverable and accessible petroleum is over, and geologically complex reservoirs are turning to be the most important sources of petroleum. These reservoirs are typically heterogeneous with significant spatial variations in lithology^1^. Therefore, improving the methods of reservoir characterization using geophysical data can be beneficial for exploration and production. However, this challenge is not easy due to the general inefficiency of geophysical inverse problems within the framework of information theory^2^. The challenge in characterizing such reservoirs is to deal with accurate inter-well correlation of major units, while simultaneously delineating subtle details for enhanced oil recovery and reservoir management. Furthermore, reservoir property estimation is usually dependent on seismic inversions, which include inherent ambiguity due to limitations linked with discrete data sampling locations, noise contamination, and modeling imperfections. Additionally, even though seismic attributes have been used in some form of predictive capacity in reservoir characterization and are common in most commercial software, a fact often ignored is whether there is a physical relationship between the seismic elastic properties and rock properties.

Over the past decades, advances in data-based predictive statistics for pattern recognition have created new approaches to constraining reservoir property estimation problems^3^. For instance, previous researchers have established that extracted seismic attribute information^4^ can be used as input features to predict and characterize hydrocarbon reservoirs via predictive neural network transforms^5–7^. In a typical workflow, a data-driven statistical transform correlates seismic data with well-logging data, recognizing the need for seismic attributes corresponding to relevant geologic features of interest^8^. Seismic attributes have also been utilized qualitatively to characterize depositional environments^9^. New developments in attribute technology such as complex trace attributes^10^, response attributes^11^, and coherence attributes^12^ have become an integral part of seismic interpretation workflow. They have been successfully applied in both prediction^6,13^ and facies classification^14,15^. However, the outstanding challenge is what to do in situations where only post-stack seismic volumes are available, but the relationships between compressional velocity and target reservoir properties exhibit a high degree of scattering at the borehole scale.

Recent advances in multi-modal input technology for predictions in the fields of computer vision, e-commerce, health informatics, and other advanced artificial intelligence applications may be beneficial in this regard^16–21^. The availability of large amounts of data and computing resources have resulted in the resurgence of data-driven machine learning techniques for the problems where conventional physics-based modeling is deficient^20,21^. For example, Moreb et al.^19^ used real-world data from a hospital in Palestine to apply a new framework that combined software engineering and machine learning for predicting health informatics. Another notable example is the assessment of seismic hazards, where the challenging problem of pinpointing small earthquakes (M_L_ < 3.0) was addressed with a convolutional network that distinguished between certain events in a target zone^22^. Extensive experiments have further demonstrated that multimodal learning using visual images and remote sensing data can perform more accurate classification of large-scale bathymetric maps^23^. Basically, the additional modalities act as constraints on the prediction mechanism. In reservoir characterization, multi-attribute transforms were employed in both linear and nonlinear modes with stratigraphic constraints to improve the predictability of reservoir properties in the Bacon Field^24^. It has also been shown that acoustic and density well logs can be applied as constraints in predicting porosity from 3D seismic data^25^. Machine learning transforms such as linear weighted fusion, support vector machines (SVMs), Bayesian inference, artificial neural networks^26^, and Kalman filters can be used to drive the fusion of input features^27^.

In this paper, we proposed to apply a new algorithm called multi-modal machine learning fusion (MMMLF) to reservoir characterization in the Bohai Bay Basin in Northeast China. The basin is a petroleum province containing fluvio–deltaic continental deposits with complex reservoirs^28,29^. The Bohai Bay Basin is one of the largest and continuously explored regions in China^30^.

## Results

### Geological characterization and data

**Figure 1:**
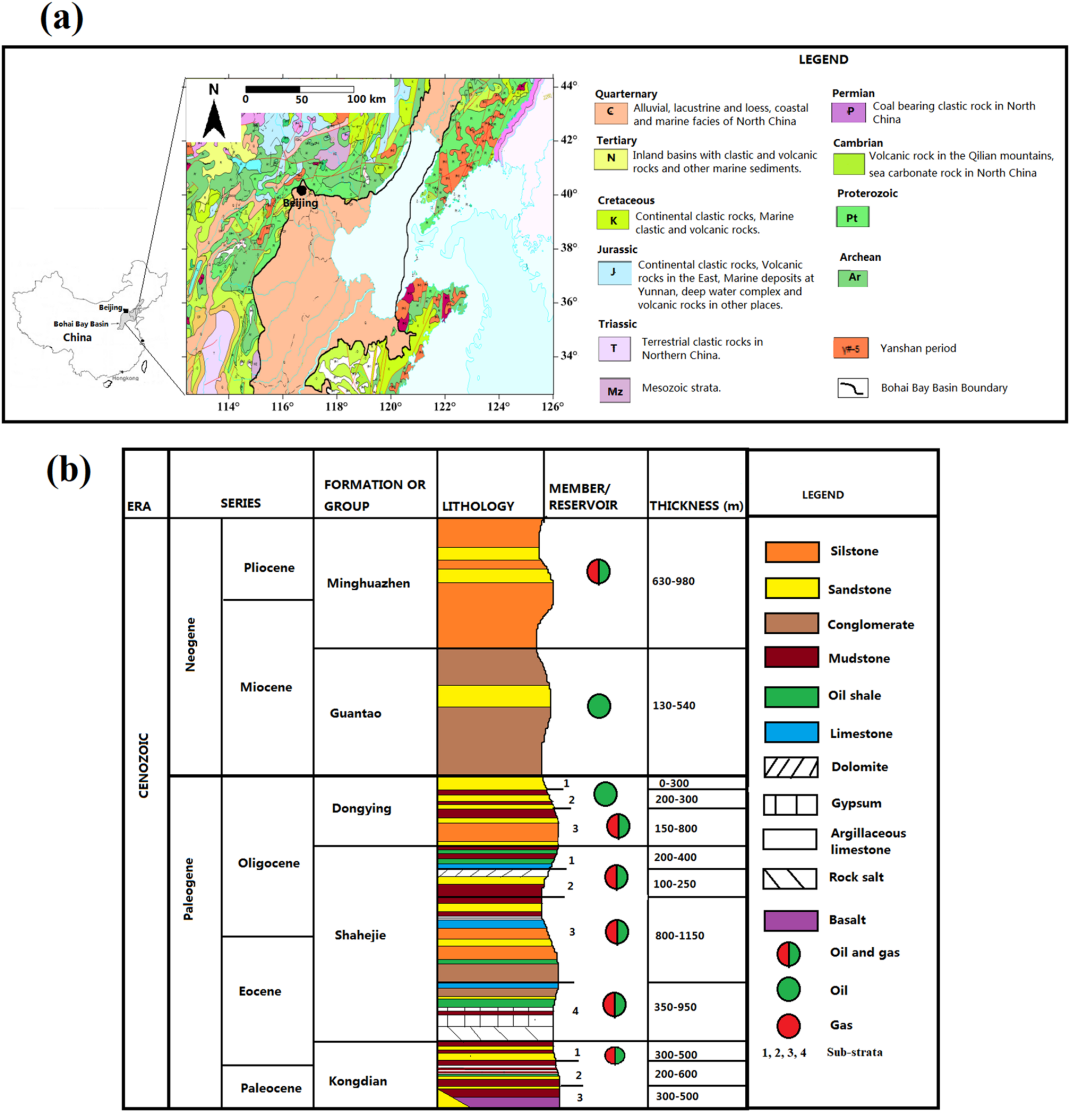
(**a**) Geological map of the Bohai Bay Basin in Northeast China and (**b**) schematic cross section of the stratigraphy of the study area. (Modified from Boateng et al.^32^).

The Bohai Bay Basin is a prolific petroleum province in Northeast China, consisting of various rift-controlled sub-basins. Figure 1 shows the geological map of the Bohai Bay Basin in Northeast China and a schematic cross-sectional view of the study area. The map in Fig. 1a shows the locations and boundaries of basins and deposits of different geological periods. Petroleum source rocks are the Paleogene lacustrine black shale and mudstone unit of Shahejie Formation^30^. Conversely, the main reservoir rocks of the system consist of the Paleogene and Neogene sandstones of nonmarine origin, clearly interbedded with lacustrine black shale and mudstone source rocks^30,31^. Sandstone reservoirs are deposited in the form of deltaic and fluvial sequences adjacent to the lakes in the center of sub-basins, and in the form of turbidite sequences in the central parts of the lakes. In this paper, we focused on the lithostratigraphic traps present in the study area, which consist of facies change, unconformity, and stratigraphic onlap varieties. They are complex and difficult to delineate. These traps were formed by the end of the Eocene, but optimal trapping conditions were probably not established until the lateral and top seals were deeply buried during the Oligocene.

Figure 1b shows various formations, lithology, and the substrata containing oil or gas. The target reservoir is a sandstone layer included in the Upper Guantao strata. The primary sandstones are characteristically braided fluvial sandstones. The lithologies of the formations included conglomerates, sandstones, and mudstones. Their porosity values ranged from 30 to 32%. The data available for analysis were borehole logs and post-stack 3D seismic volumes. We use gamma ray logs to represent lithology. The most easily accessible structural faults and anticlinal traps previously explored are in production and are approaching maturity. Discovering new oil and gas reservoirs requires delineating subtle stratigraphic traps.

**Figure 2:**
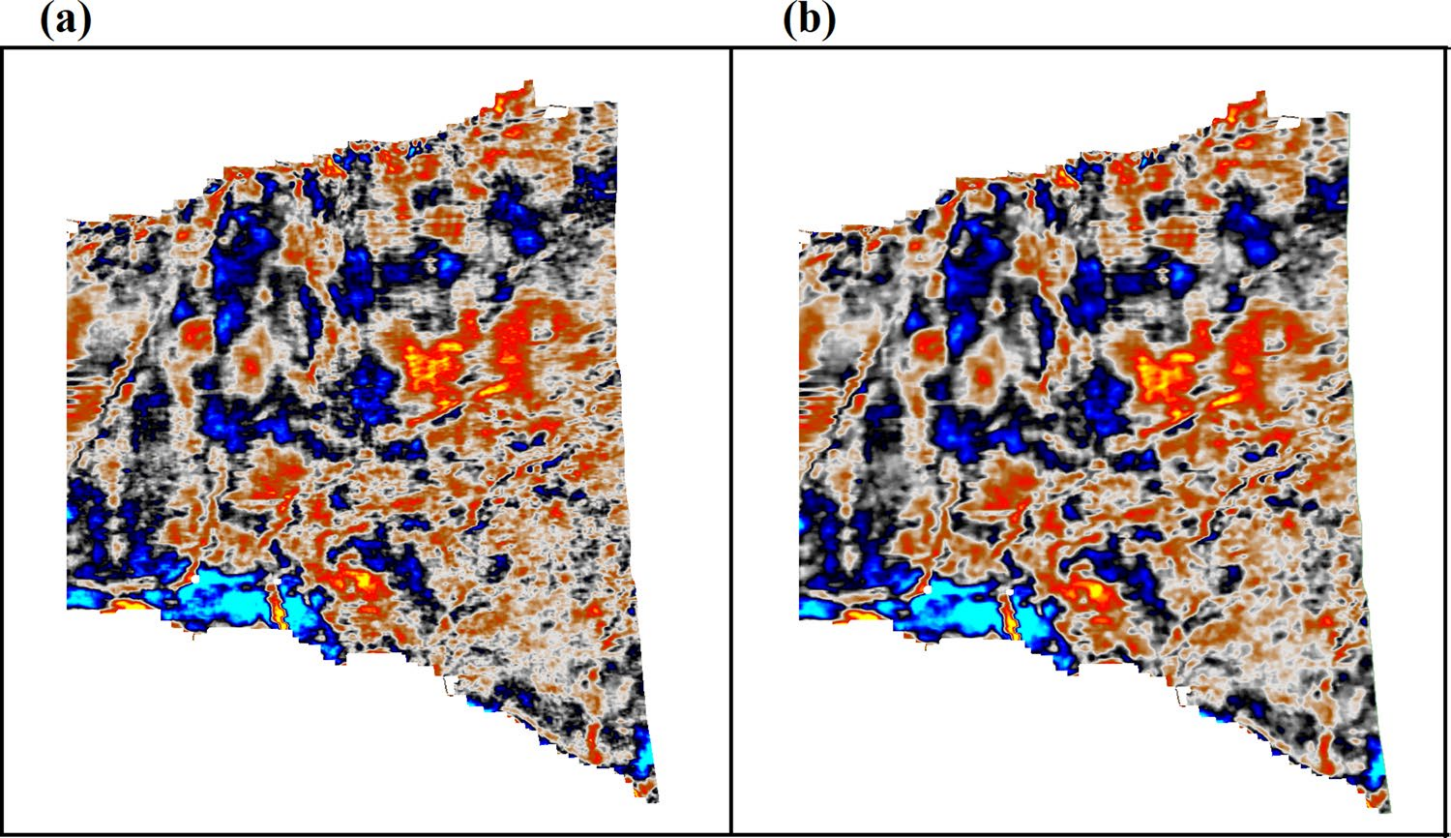
Effects of seismic filtering algorithm in (**a**) raw seismic profile and (**b**) filtered seismic profile.

However, before applying the seismic data to the delineation of subsurface features, it is essential that the data be sufficiently noise-free. This was done to ensure that the signal-to-noise ratio of the seismic data is adequate and that the seismic response predominantly reflects the sediments in the target area. For this purpose, we applied an advanced post-stack processing algorithm. Figure 2 shows the effect of the filtering algorithm. The filtered and unfiltered seismic volumes are compared in Fig. 2a,b. An improvement in the continuity and clarity of the filtered seismic volumes was observed. Therefore, the signal-to-noise ratio (SNR) of the seismic data was improved.

### Seismic attributes and structural characterization

**Figure 3:**
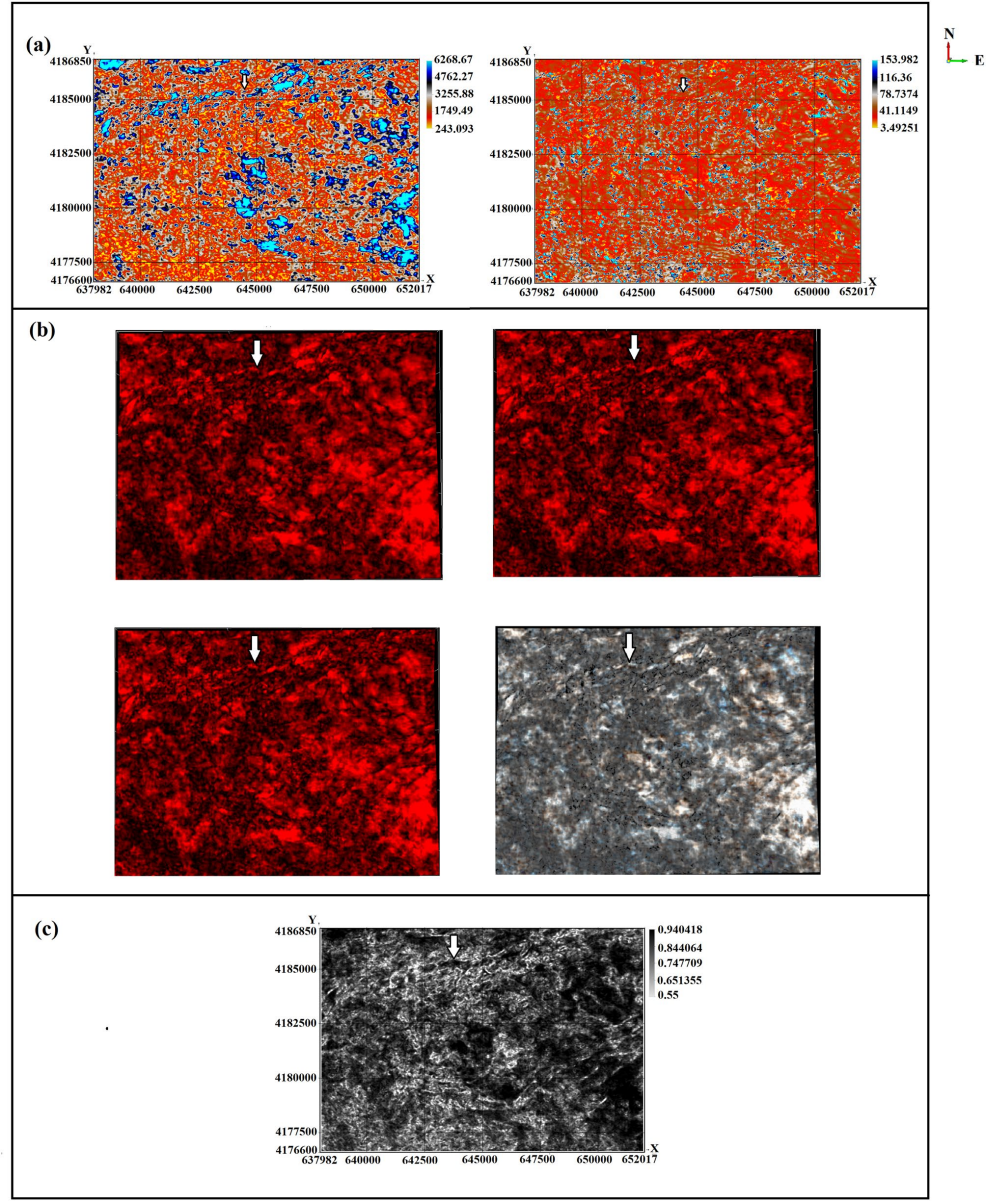
Seismic attributes extracted along the Upper Guantao layer: (**a**) instantaneous amplitude and instantaneous frequency, (**b**) spectral decomposition attributes (upper left: 30 Hz iso-frequency map; upper right: 40 Hz iso-frequency map; bottom left: 50 Hz iso-frequency map; bottom right: RGB blended horizon map), and (**c**) similarity attribute.

Seismic attributes, mathematical transforms of seismic data^9^, were used to characterize the stratigraphic features of the Upper Guantao sub-formation. For example, complex trace attributes^10^ have been used to highlight lithological changes. Figure 3a shows the complex trace attributes of instantaneous amplitude and frequency. The instantaneous amplitude attribute measures the reflection strength and indicates the depositional environment. In the figure, the high-amplitude zones are interpreted as braided fluvial sandstones interbedded as lenses. Therefore, changes in amplitude are correlated with lithological changes. The image at the bottom of Fig. 3a shows the instantaneous frequency attribute that can be used as an indicator of bed thickness. Here, high frequencies are interpreted as thinly-laminated shales, while low frequencies indicate massive bedding geometries, such as sand prone lithologies. The consistency of high-amplitude and low-frequency zones enhanced the interpretation of braided fluvial sandstone.

To better understand the extent and distribution of the fluvial sandstones, we took advantage of the spectral decomposition attributes. It is an efficient tool for identifying paleo-geomorphological features and revealing stratigraphic information along an interpreted horizon^33^. This method transforms the seismic data into the time–frequency domain using the Fourier transform technique to show the sand thickness by accentuating the channel features. Three iso-frequency bands of 30, 40, and 50 Hz were extracted and combined with Red Green Blue (RGB) blending. Figure 3b shows the iso-frequency band maps and RGB blended layer map along the Upper Guantao layer. Tuning cube frequencies of 30, 40, and 50 Hz captured the subtle physical changes and revealed depositional features. High-amplitude regions appeared as bright zones with channel-like shapes on the spectral decomposition maps. The sand channels can be clearly identified in the 30, 40, and 50 Hz iso-frequency maps. In the RGB blended image (bottom right of Fig. 3b), a complex system of channels at the top of the map shows a trend from southwest to northeast. The location of the channel complex was consistent with the interpretation of braided fluvial sandstones as sand lenses. Finally, the similarity attribute was also applied to the Upper Guantao layer to further understand the spatial distribution of fluvial sands and discontinuity boundaries in the study area. Similarity is a post-stack seismic attribute that returns the trace-to-trace similarity properties. Also known as coherence, it shows to what extent two or more traces are completely identical in waveform and amplitude^12^. Stratigraphic boundaries are associated with low similarity values. Figure 3c shows the Upper Guantao layer similarity attributes. The discontinuity boundaries of the channel complex were clearer and enhanced on the similarity attribute image. Moreover, the previously observed sandstone lenses were clearly identified by complex trace and spectral decomposition attributes.

### Rock physics trends

**Figure 4:**
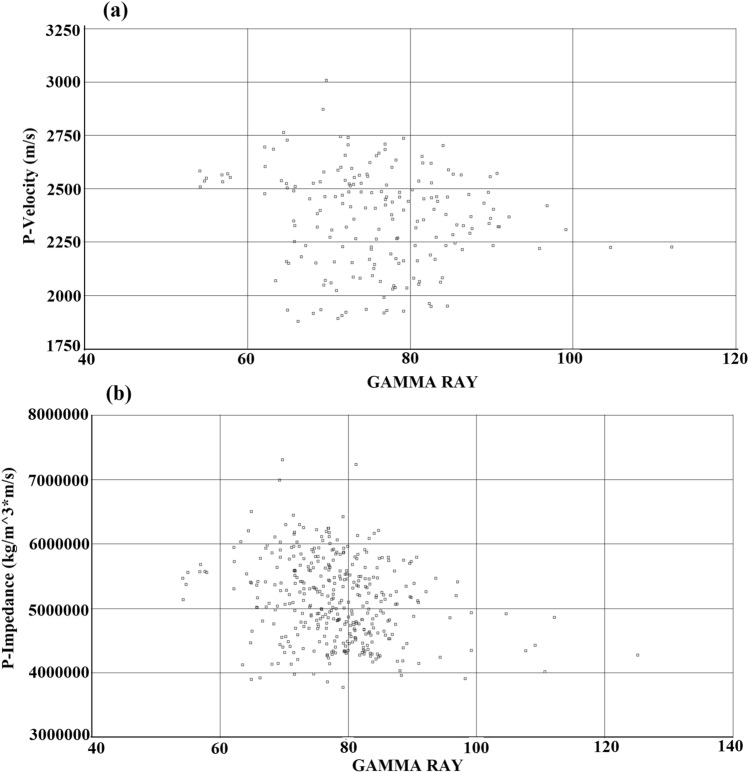
(a) P-velocity versus gamma ray for Wells X1 and X2 and (b) P-impedance versus gamma ray for Wells X1 and X2.

The seismic reflection response depends on the acoustic impedance contrasts between subsurface layers. To determine lithology and reservoir parameters from post-stack seismic data, a complete understanding of the relationship between lithological logs and acoustic properties is required. In the study area, there were two boreholes (X1 and X2) with acoustic logs, acoustic impedance, and gamma ray logs that represented lithology. The two boreholes were the sampling locations where the data was sampled vertically. Cross-plots of acoustic velocity against gamma ray and acoustic impedance against gamma ray for the Upper Guantao layer are shown in Fig. 4a,b, respectively. In Fig. 4a, there is an inverse relationship between p-velocity and gamma ray (i.e., negative correlation coefficient). High gamma ray shales clustered at low velocities. Dry fluvial sandstones with moderate gamma ray values clustered around intermediate velocities, while wet fluvial sandstones had higher p-velocities. Therefore, acoustic velocity can identify sandstone reservoirs, although there is uncertainty due to large data scattering. Figure 4b shows a plot between acoustic impedance and gamma ray in the Upper Guantao layer, using data from Wells X1 and X2. The plot indicates an inverse relationship (i.e., negative correlation coefficient) between gamma ray and acoustic properties. Large scattering between the acoustic properties of the formation and the gamma ray logs can lead to estimation ambiguity in the predictive algorithm. However, based on the observations from cross-plots, seismic attributes calculated from post-stack seismic volumes can predict the lithological properties.

### Lithological prediction by multi-modal machine learning fusion (MMMLF)

In this section, the spatial distribution of lithology is determined by running a gamma ray prediction using MMMLF. Details are provided in the Materials and Methodology section. Input features are different modes of seismic data that are extracted as seismic attributes. Of the total number of input attributes generated, those relevant to prediction were seismic inversion, instantaneous amplitude, and 30 Hz iso-frequency attribute. Table 1 shows three main seismic attributes with the best correlations to gamma ray logs. The three attributes were obtained through stepwise regression. The input seismic attributes were fused in the neural network fusion engine to predict the spatial distribution of gamma rays.

**Table 1:** Three main seismic attributes with the best correlations to gamma ray logs.

Seismic attribute	RMSE
Seismic inversion	4.20
Instantaneous amplitude	6.09
30 Hz iso-frequency	7.40

The fusion mechanism was performed by using a supervised neural network to establish a nonlinear relationship between the seismic response and reservoir property of interest (gamma ray). The neural network is a fully connected multilayer perceptron with one hidden layer. Backpropagation with momentum and weight decay was utilized as a learning algorithm in the execution of the fusion engine. The workflow is explained in the Materials and Methodology section. For each well, a single composite trace was extracted from the relevant seismic attribute volume by averaging the nine nearest traces around the borehole. The gamma ray logs were converted from depth to time and sampled at the same sampling rate as the seismic data.

**Figure 5:**
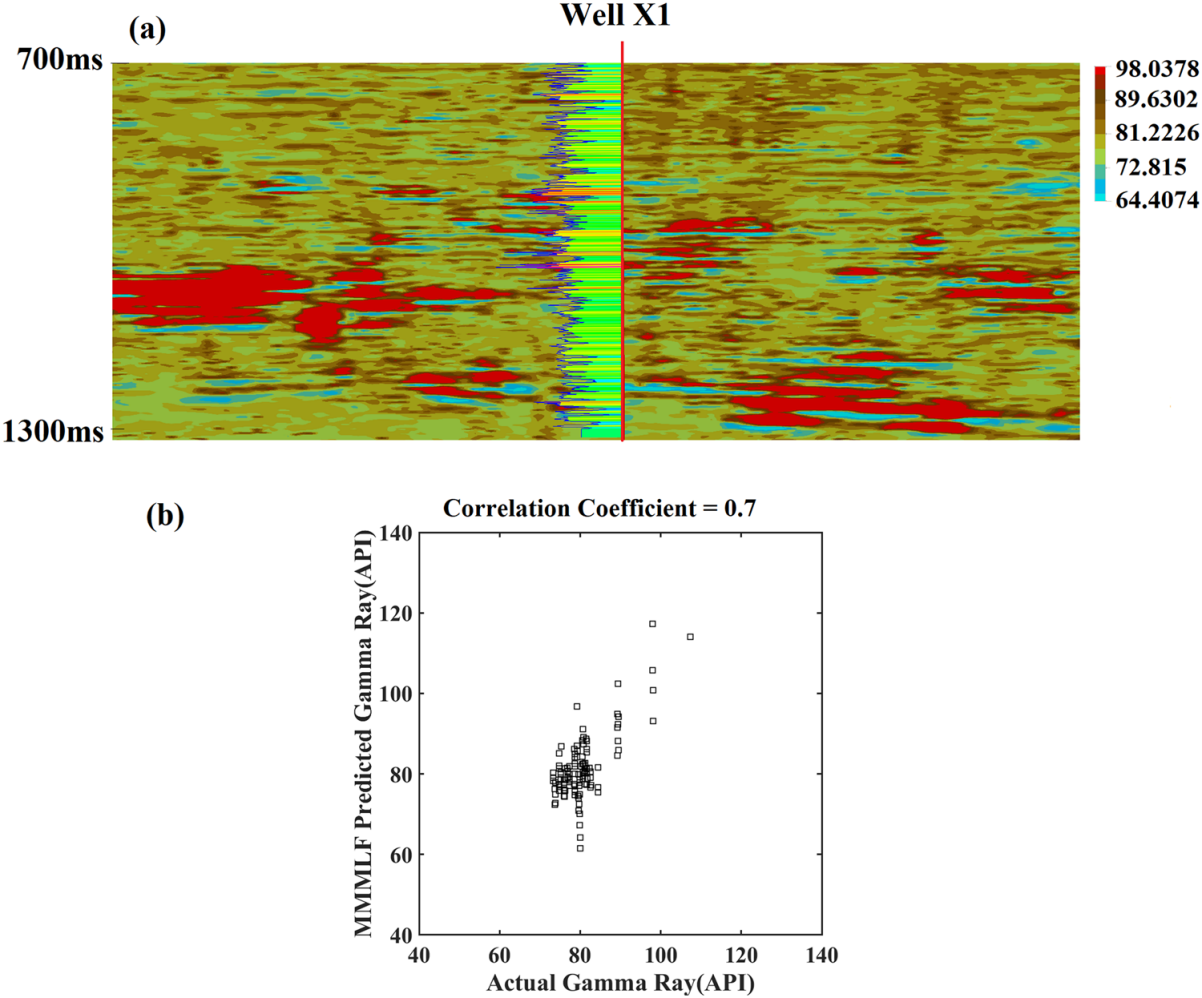
Comparing actual gamma ray and predicted gamma ray using cross-validation results: (**a**) inverted gamma ray section for random profile section intersecting Well X1 and (**b**) predicted gamma ray versus actual gamma ray for Well X1.

Finally, the learned MMMLF was applied to gamma ray inversion using the seismic attributes as input. Figure 5a shows an inverted lithological volume intersecting Well X1. This is the cross-validated result from a random profile from the inverted volume. Potential stratigraphic traps containing a combination of saturated sands (in blue color) and shale seal (in red color) are observed in the image. It was also observed that the gamma ray logs from Well X1 were in good agreement with the lithological volume. Figure 5b compares the predicted gamma ray log for Well X1 with the actual gamma ray log. The correlation coefficient between the actual gamma ray log and the predicted gamma ray log was 0.7, and the RMSE value was 5.93. The predicted gamma ray from the MMMLF algorithm shows significant correlation with the actual gamma ray given the lack of a clear relationship at borehole scale and only two boreholes as training data. Therefore, the MMMLF algorithm successfully predicted the lithological distribution in the Upper Guantao layer. It also indicated that the MMMLF algorithm was able to estimate gamma ray properties beyond the boreholes. The prediction results significantly helped to characterize the sandstone reservoirs in Northeast China. To test the robustness of the MMMLF methodology, the popular SVM algorithm was applied to the same reservoir property problem and the results are shown in Table 2. Table 2 compares the prediction results for MMMLF and SVM using the correlation coefficient and Root-Mean-Square Error (RMSE). Even though the RMSEs of the two algorithms were similar, the correlation coefficient of the MMMLF prediction was higher than the SVM prediction.

**Table 2:** A comparison of prediction results for MMMLF and SVM algorithm results in terms of the correlation coefficients and RMSEs.

ML algorithm	R	RMSE
MMMLF	0.7	5.93
SVM	0.5	5.33

**Figure 6:**
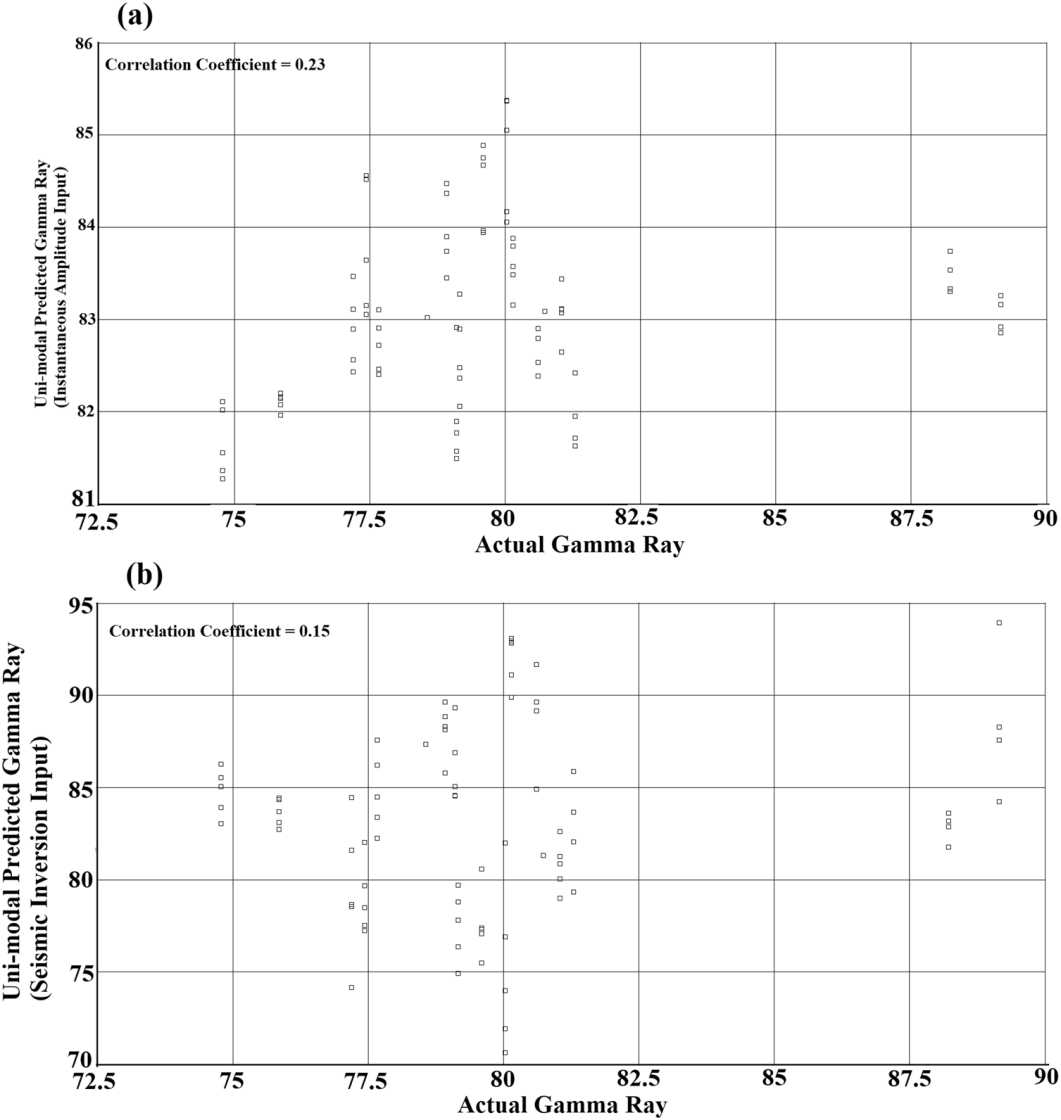
(**a**) Predicted gamma ray from instantaneous amplitude input versus actual gamma ray for Well X1 and (**b**) predicted gamma ray from seismic inversion input versus actual gamma ray for Well X1.

One of the advantages of utilizing multi-modal inputs over the single inputs in machine learning predictive algorithms is the superadditive effect. To observe this effect, we attempted to use individual seismic attributes in the prediction algorithm. This essentially simulates uni-modal prediction. The individual seismic attributes consist of instantaneous amplitude attribute and seismic inversion attribute. Uni-modal predictions were performed using individual seismic attributes and the results are shown in Fig. 6. Figure 6a shows the predicted gamma ray from the instantaneous amplitude input versus actual gamma ray. Figure 6b shows the predicted gamma ray from the seismic inversion input versus actual gamma ray for Well X1. The correlation coefficients from the predicted gamma ray logs were 0.23 and 0.15 for the instantaneous amplitude input and the seismic inversion input, respectively. It was understood that none of the single modes could successfully predict the lithological distribution independently, leading to poor predictions. Therefore, comparing the multi-modal and uni-modal prediction algorithms in this case study, it can be said that MMMLF enhances the ability of the fusion engine to predict due to the superadditive effect.

## Discussion and conclusion

We proposed a multi-modal machine learning fusion to predict the spatial distribution of reservoir properties and applied the method to delineate a fluvio–deltaic sandstone reservoir and stratigraphic traps in an oilfield in the Bohai Bay Basin, Northeast China. Our findings suggested that the multi-modal machine learning fusion technique enhanced the prediction of lithological distribution in the Upper Guantao layer. Furthermore, our results showed that multi-modal inputs were more effective in prediction algorithms than uni-modal input processes. The process captures multi-modal sensory information from the subsurface in a bio-integrated framework for intermediate-level decisions that can provide complementary information. Furthermore, the accuracy of the final prediction is improved. Petrophysical properties are difficult to predict, and sufficient input features are required to limit the number of degrees of freedom, especially in complex fluvio-deltaic environments. Our findings correspond to previous results^6^ regarding the effectiveness of multi-modal approach in predicting reservoir properties for characterizing a reservoir. In certain case studies, uni-modal inputs were adequate for predicting reservoir properties. In a case study^34^, acoustic impedance was used to predict the porosity of Eagle Ford shale. We consider these results reasonable given the study area (144.11 km^2^) covered by the data and the algorithm was trained on data from two wells. To further improve the results in future studies, additional sampling locations in the form of boreholes are required for training the machine learning algorithm. Nevertheless, this study shows that the MMMLF algorithm is a promising method for processing large amounts of data in oilfields and identifying potential oil pools for production, which is a challenging task in petroleum exploration. The limitation of this method is selecting a representative depth interval for training the dataset. This should be done with caution as the machine learning algorithm fails to make accurate predictions if the relevant depth section is not selected. Therefore, in the preprocessing step, the target interval must be carefully selected. Furthermore, if the number of independent well measurements is small^35^, the probability of observing false sample correlations between seismic attributes and well data can be high. To implement our algorithm, cross-validation was applied to ensure there were no false correlations, as shown in Fig. 5. We also demonstrated the superadditive effect, which suggests that multi-modal input predictions perform better than uni-modal input predictions.

In this application, there are three layers in the neural network fusion engine used in the multi-modal machine learning fusion algorithm. The relationship between seismic responses and reservoir properties is nonlinear and complex, but neural networks as fusion engines can deal with that. The MMMLF methodology proposed in this case study successfully addressed the challenge of using data-based machine learning predictive tools when there is large scattering between acoustic and target reservoir properties. Machine learning has become very influential in seismic reservoir characterization because it enhances seismic interpretation and the identification of productive zones in petroleum provinces, especially in estimating and predicting reservoir properties. In ideal situations, the relationship between rock properties is clear and there is a direct basis for inversion. However, in the case of a high degree of scattering, it is very difficult to run accurate petrophysical inversions. In complex fluvio–deltaic environments, this challenge is often severe and requires careful consideration. Therefore, machine learning data-based tools for predicting reservoir properties are effective.

It has been demonstrated that after appropriate advanced post-stack filtering of the seismic data, relevant seismic attributes and borehole data can be fused in a multi-modal machine learning fusion to predict lithology distribution based on gamma ray logs at the Bohai Bay Basin, Northeast China. The conclusions from this study can be summarized as follows. First, reservoirs in the study area consisted of facies change, unconformity, and stratigraphic traps. Second, a dip-steered median filter accessed dip and azimuth information from seismic data and improved the continuity and clarity by increasing the SNR ratio. Third, the acoustic properties were inversely proportional to the lithological properties of the Upper Guantao layer in the study area. Finally, multi-modal machine learning fusion successfully predicted stratigraphic reservoirs in the Upper Guantao layer of the Bohai Bay Basin study area. These findings may have significant implications for future exploration and production of the remaining resources in the Bohai Bay Basin of China and similar petroleum provinces around the world. The framework also serves as a platform for using advanced data-based predictive machine learning tools with other geophysical methods.

## Materials and methodology

The Bohai Bay Basin has been explored and produced oil for decades. In this basin, oil and gas reservoirs are located at different depths. This study focused on the depth intervals to cover the entire Upper Guantao layer. Reservoir property prediction is a unique problem in oil and gas exploration that has been well studied^4,6,13^. The ability to accurately predict the spatial distribution of reservoir properties is essential for exploiting petroleum resources. Borehole gamma ray log measurements were used to represent lithological variations and predict the distribution of the fluvio–deltaic sandstones.

### Dataset and filtering

The data used in this study was from seismic responses to subsurface impedance variations and borehole logs. In this study, we assumed that the relevant depth sections were already identified and seismic data and borehole logs were extracted. The seismic data consisted of 560 inlines and 410 crosslines, with a total of approximately 250,000 traces sampled every 2 ms. The total survey area was about 144.11 km^2^. We also assumed that the relevant seismic section has been matched to the borehole depth through a well-to-seismic tie. Well logs are direct high-resolution vertical measurements acquired at specific boreholes in the study area. Conversely, seismic data is recorded spatially, has a lower resolution and is in the time domain. Well-to-seismic tie is a significant process in reservoir characterization workflows and has been studied severally^36,37^. The seismic data was used as input data for prediction, while the gamma ray logs showed the properties of the target reservoir. In each well, the gamma ray logs were converted to the time domain and seismic attribute traces were extracted near the borehole to train the machine learning algorithm.

Oil and gas reservoir rocks have emerged as a result of fluvial and deltaic depositional processes^30^. Fluvio–deltaic deposits often exhibit small-scale lateral variations which generate noisy artifacts in the processed seismic sections^38^. Additionally, due to the limited resolution of seismic methods, the geological complexity of stratigraphic reservoirs is poorly imaged. To significantly improve the resolution, unrealistic high frequencies must be recorded^39^. Since this is a difficult process, filtering the processed seismic data was selected as an alternative. Before interpreting seismic data, the data must be sufficiently free of noise. This is to ensure that the seismic response mainly reflects the sedimentary strata in the area of interest^40^. Although a good signal-to-noise ratio (SNR) depends heavily on careful acquisition and processing of seismic data, remnants of noise can mask the subsurface characteristics of the processed sections. These residual noisy artifacts can be removed by performing advanced post-stack processing on the seismic data. This algorithm enhances the data with azimuth and dip-oriented filtering to increase resolution and remove incoherent noise. This process is also known as dip-steered based filtering^41^. Dip calculations are then performed^42^. In this method, the 3D seismic signal can be written as:

1 $$u\left( {x,y,t} \right) \quad x,y,t \in Z$$

Next, a Fourier transform is performed on the whole signal and the signal is multiplied by the window function (i.e., the signal is transformed into *f-k* domain by the discrete Fourier transform). The dip is then calculated using the Radon spectrum as a weighting function for the dips in the *x* and *y* directions:

2 $$\overline{p}_{x} = \frac{{\iint {p_{x} \overset{\lower0.5em\hbox{$\smash{\scriptscriptstyle\smile}$}}{P} \left( {p_{x} ,p_{y} } \right)dp_{y} dp_{x} }}}{{\iint {\overset{\lower0.5em\hbox{$\smash{\scriptscriptstyle\smile}$}}{P} \left( {p_{x} ,p_{y} } \right)dp_{y} dp_{x} }}}$$

3 $$\overline{p}_{y} = \frac{{\iint {p_{y} \overset{\lower0.5em\hbox{$\smash{\scriptscriptstyle\smile}$}}{P} \left( {p_{x} ,p_{y} } \right)dp_{x} dp_{y} }}}{{\iint {\overset{\lower0.5em\hbox{$\smash{\scriptscriptstyle\smile}$}}{P} \left( {p_{x} ,p_{y} } \right)dp_{y} dp_{x} }}}$$

where $\overline{p}_{x}$ and $\overline{p}_{y}$ are the average dips in the *x* and *y* directions, respectively. For each position, the dip and azimuth corresponding to the local maxima of a third-order 3D polynomial that fits the subcube around the highest energy sample in the Fourier domain is used as output^43^. The information of the dip and azimuth is then stored in the steering cube. The dip-steered median filter then processes the data by accessing the dip and azimuth information stored in the steering cube.

### Multi-modal machine learning fusion (MMMLF) methodology

In biological systems, sensory perception for effective interpretation and understanding is multi-modal^44^. However, remote sensing instruments such as seismic acquisition equipment that help us understand subsurface features are uni-modal sensory instruments. Biological system studies have shown that multi-modal perceptions have clear advantages over uni-modal perceptions. One such advantage is the superadditive effect of multisensory integration^45^. Superadditivity is a situation where the total multisensory is greater than the sum of its unisensory parts. Mathematically, the multi-modal effects can be described linearly as follows:

4 $$y_{ij} = \mathop \sum \limits_{r = 1}^{N} a_{ir} x_{jr}$$

where *i* = 1,…*I* and *j* = 1,…*J*. Equation (4) is interpreted as *y*_*ij*_ being the linear combination of *N* signals *x*_*j*1_,…,*x*_*jN*_ impinging on sensor *i* at sample index *j*, with weights *a*_*i*1_,…,*a*_*iN*_. However, in most cases, the relationships between natural system properties such as subsurface data are not linear. To effectively use the multi-modal effects in reservoir characterization, it is necessary to develop a nonlinear formulation as a fusion engine. An example of an efficient data-driven algorithm that can fuse different modalities is a neural network. The mathematical expression of biological neurons can be written as an activation function (*A*). The sigmoid function [Eq. (5)] is widely used as an activation function:

5 $$A\left( W \right) = \frac{2}{{1 + exp\left( { - W} \right)}} - 1$$

For reservoir characterization, different modes of data are available for interpretation such as, petrophysical logs and seismic data. In the present investigation, the borehole logs and seismic data of the study area contained the relevant subsurface information. However, the measured physical properties depend on different types of instruments, measurement techniques, experimental setups, and processing procedures. Integrating different types of data favors geological interpretations. For analysis purposes, seismic data is decomposed into its components^10^ to take advantage of multi-modal machine learning fusion. These components were in the form of various seismic attributes. Several attributes can be derived from the seismic volume. Seismic attributes were adopted as input features and borehole logs related to lithological properties deployed as expected output features.

The performance of machine learning algorithms depends heavily on finding a suitable feature representation space. The relevant multi-modal features were defined as inputs corresponding to different geological features of interest in the target interval. These relevant features were obtained by extracting seismic attributes from the post-stack seismic data, defined as “any measure of seismic data that helps us visually enhance or quantify the features being interpreted”^41,46^. In this study, seismic attributes extracted from the filtered 3D volume were instantaneous amplitude, instantaneous frequency, different iso-frequency bands, spectral decomposition, similarity, and acoustic impedance. We used a simple feature selection approach to find a suitable feature representation space. Feature selection aimed to select relevant feature subsets from the original set of seismic attributes that were able to efficiently describe the intrinsic characteristics of the input data. And it is executed by reducing the impact of noise and eliminating irrelevant features^47^. Moreover, feature selection allowed us to avoid computing in high dimensions by reducing the input space and only searching for relevant attributes with significant relationships to the reservoir characteristics of interest. This was achieved by using stepwise regression^6^.

For multimodal input features, Eq. (4) can be rewritten in the following form:

6 $$P\left( {x,y,z} \right) = f[M_{1} \left( {x,y,z} \right) + M_{2} \left( {x,y,z} \right), \ldots , + M_{m} \left( {x,y,z} \right)]$$

where *f* is a nonlinear function, *P* is the target petrophysical property of interest, and *M*_1_ to *M*_*m*_ are different modes of the input data. The nonlinear function represents the fusion engine, and in this paper, an artificial neural network was used for this purpose.

MMMLF is updated using a traditional backpropagation algorithm to detect nonlinear relationships between data specific to a reservoir. The popular backpropagation technique^48^ features a least-squares algorithm and is the simplest version of all steepest descent optimization methods. The cost function for this problem was defined as the following mean squared error performance function:

7 $$E = \frac{1}{2}\mathop \sum \limits_{k} \mathop \sum \limits_{t} e_{k}^{2} \left( t \right) = \frac{1}{2}\mathop \sum \limits_{k} \mathop \sum \limits_{t} \left[ {d_{k} \left( t \right) - o_{k} \left( t \right)} \right]^{2} ,$$

where *d*_*k*_(*t*) is the desired output and *o*_*k*_(*t*) is the actual output from the algorithm. Applying the backpropagation learning algorithm to reduce the cost function leads to the updated MMMLF equation.

**Figure 7:**
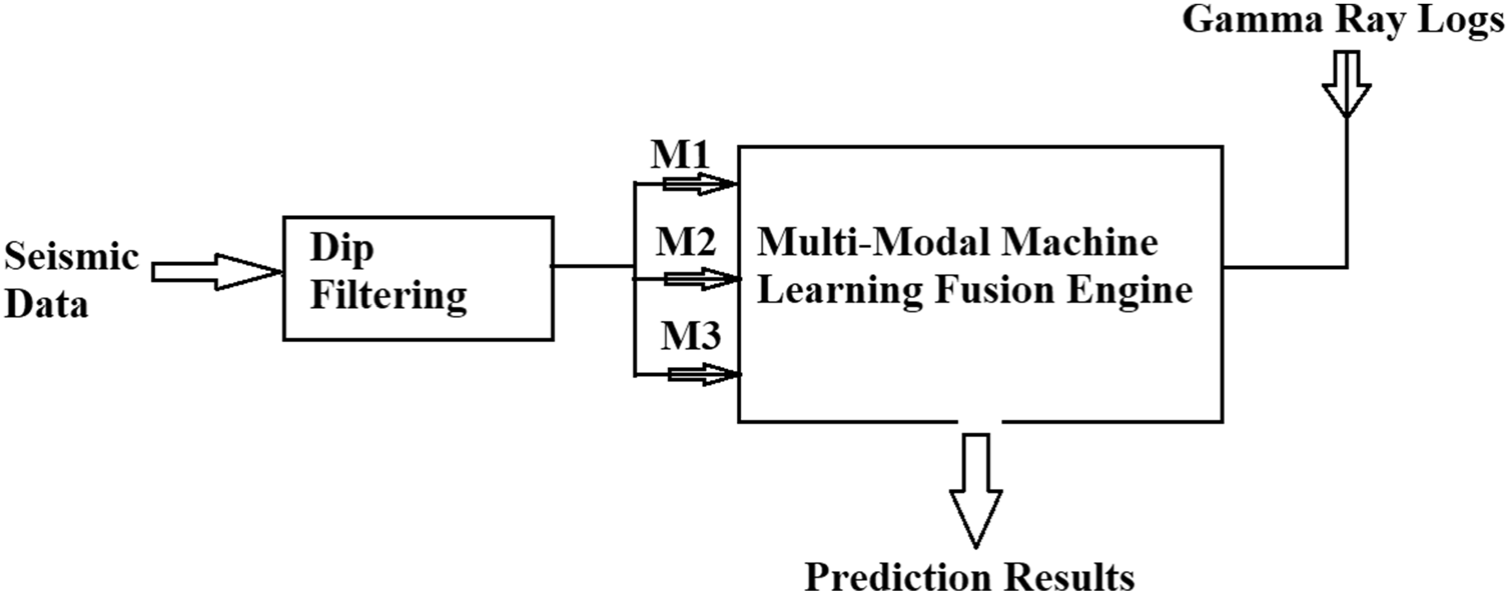
Workflow of multi-modal machine learning fusion (MMMLF).

Fusing multi-modal features through capturing intermediate-level decisions provides additional information and improves the accuracy of the final prediction process. This methodology was an alternative approach for cases where (1) it was difficult to identify direct relationships between the seismic attributes and rock properties from the physical principles, and (2) there was a relationship between the acoustic velocity and the lithological properties of the target reservoir at the borehole scale but the relationship included extensive scattering due to heterogeneity. In the former example, a single mode extracted from post-stack seismic data can provide a data-based relationship for predicting reservoir properties. However, in the latter, a constraint is required for predictions and hence the introduction of complementary modes to constrain the scatter in the data through an intermediate decision. To convert seismic attributes to lithological properties, rock physics crossplots were used to validate the relationships between lithology and acoustic wave properties. The workflow of multi-modal machine learning fusion is schematically shown in Fig. 7. The algorithm used in this study was written in MATLAB.

Systematic cross-validation tests of the data were developed^49^ to analyze the accuracy of the MMMLF technique. This was done by removing one well from the training dataset. MMMLF was trained on the remaining well and applied to seismic traces of the hidden well location. Next, the MMMLF output was compared to the actual gamma ray log. Finally, the test was repeated for each well in the training dataset. Cross-validation offered an excellent technique to validate the accuracy of the method.
